# Novel Biomarkers for Predicting Preeclampsia

**DOI:** 10.1016/j.tcm.2008.07.002

**Published:** 2008-07

**Authors:** David M. Carty, Christian Delles, Anna F. Dominiczak

**Affiliations:** BHF Glasgow Cardiovascular Research Centre, University of Glasgow, G12 8TA Glasgow, United Kingdom

## Abstract

Preeclampsia is a major cause of maternal morbidity and mortality worldwide. Despite decades of research into the condition, the ability of clinicians to predict preeclampsia prior to the onset of symptoms has not improved significantly. In this review, we will examine the pathophysiology underlying preeclampsia and will look at potential biomarkers for early prediction and diagnosis. In addition, we will explore potential future areas of research into the condition.

## Introduction

Preeclampsia is a multisystem disorder of pregnancy, which complicates 3%-5% of pregnancies in the western world. It is a major cause of maternal morbidity and mortality worldwide. The cardinal clinical features of the condition are hypertension and proteinuria occurring after 20 weeks gestation in women who were not previously known to be hypertensive. Other signs and symptoms include edema and headache, and in severe cases, the condition is associated with seizures (eclampsia), liver, and kidney dysfunction as well as clotting abnormalities, Adult Respiratory Distress Syndrome and fetal growth restriction (FGR) (Davison et al. 2004).

The cause of preeclampsia remains unknown, and the only known cure is delivery of the fetus and placenta. Various agents have been evaluated to see whether they influence the development of preeclampsia. Aspirin has been studied extensively; it was summarized in a Cochrane review by Duley et al. (2004) to have modest but significant benefits in preventing preeclampsia. It is commonly used in women who are known on the basis of risk factors to be at high risk of developing preeclampsia, although questions remain over exactly which subgroups of women are most likely to benefit. Studies of calcium supplementation suggest that it is of most benefit when used in high-risk women with low dietary calcium intake (Hofmeyr et al. 2006), although studies of antioxidants such as vitamins C and E and magnesium and zinc supplements have been less promising (Poston et al. 2006).

Despite decades of research into the condition, predicting which women are at increased risk of developing preeclampsia remains problematic. Identifying “at-risk” women is an important aim; because modern obstetric care places emphasis upon the primary care setting for expectant women, a marker which identified high-risk women would allow for closer supervision in secondary care. Such a marker would also facilitate recruitment for trials of potential therapeutic agents, for accurate diagnosis, and for timely intervention whenever problems develop. Furthermore, predicting preeclampsia in women with underlying conditions such as diabetes and chronic hypertension would be of great clinical value.

**Figure 1:**
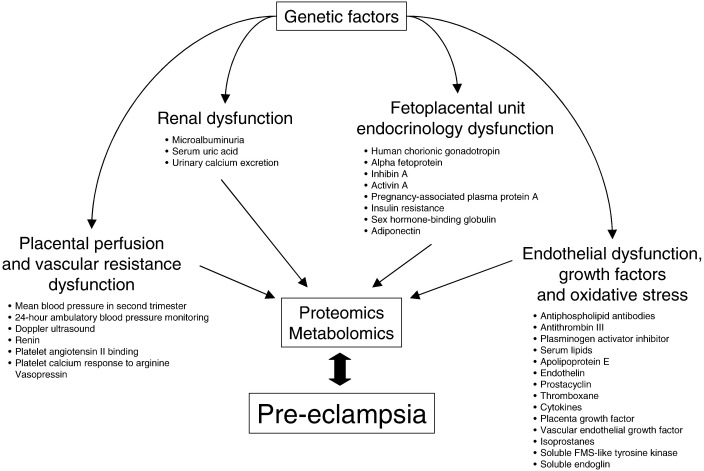
Biomarkers of pre-eclampsia. Biomarkers of preeclampsia are grouped into four major categories (modified from data from Conde-Agudelo et al. 2004). Production and levels of biomarkers are ultimately dependent on genetic factors and therefore genomic studies are likely to detect genetic variants associated with preeclampsia. However, in contrast to the static genome, the proteome is dynamic. Whereas the genome will not change during pregnancy or pregnancy-associated conditions such as preeclampsia, the proteome will change. This is indicated by the double-headed arrow. Proteomic and metabolomic studies will therefore reflect a large number of biomarkers and their actual levels and will more accurately predict risk than genomic studies.

Clinicians have traditionally relied on maternal risk factors, such as increased maternal age, family history, and preexisting diseases, for determining which women are at increased risk. The problem with using these risk factors is that millions of women worldwide have these risk factors but do not develop preeclampsia. Moreover, the majority of them are nonmodifiable. There have been many screening tests evaluated in the literature over the years for predicting preeclampsia; these have been comprehensively reviewed in a World Health Organization publication (Conde-Agudelo et al. 2004) (Figure 1). A further summary of predictive tests, including a review of preventative interventions and economic modeling, has been recently published by the UK National Institute for Health Research (Meads et al. 2008).

The imaging technique that has so far been most widely used for predicting preeclampsia has been uteroplacental Doppler ultrasound. Impaired placental perfusion, one of the hallmarks of preeclampsia, can be assessed by measuring flow waveform ratios or by detecting diastolic notching of the uterine arcuate vessels. Uterine Doppler studies can, however, be inconsistent: different types of machine, different gestational age at assessment, and different definitions of abnormal flow velocity waveforms make accurate comparison of studies difficult. A large systematic review of 43 studies involving 40,000 patients (Conde-Agudelo et al. 2004) found that, in both low- and high-risk patient groups, the positive predictive value was not sufficiently high to recommend routine screening.

Attention has therefore turned in recent years towards identifying maternal markers of placental dysfunction which are raised in women who go on to develop preeclampsia. In this review, we will look at novel biomarkers which have been used in the prediction of preeclampsia and explore potential future areas of investigation.

## Pathogenesis of preeclampsia

Although the cause of preeclampsia remains elusive, the origin of the condition is recognized as lying in the placenta. This is known to be the case because preeclampsia occurs only in the presence of pregnancy, it resolves after delivery of the placenta and it can occur in the absence of a viable fetus, for example, in molar pregnancies. Placental development is a closely regulated process which is essential for normal fetal development and for maintaining a successful pregnancy. Blood supply to the placenta is via the spiral arteries, which, in turn, are branches of the uterine arteries. Early in normal pregnancy, the cytotrophoblastic cells of the developing placenta invade the uterine wall, disrupting the endothelium and tunica media of the spiral arteries. The vascular wall of the spiral arteries is remodeled; this, in turn, leads to a transformation of the spiral arteries from low-flow, highly resistant vessels into the high-flow, low-resistance vessels which are vital for normal placental development.

There are thought to be two stages to cytotrophoblastic invasion: the first involves invasion of the decidual segments of the spiral arteries, at around 10-12 weeks gestation; the second involves invasion of the myometrial segments at 15-16 weeks (Sargent and Smarason 1995). In preeclampsia, the cytotrophoblastic invasion of the myometrial segments is impaired: the spiral arteries remain narrow, and blood supply to the fetus is restricted. The effects of this on the fetus become more significant as pregnancy progresses, since the uterine vasculature is unable to keep up with the increased amount of blood and nutrients necessary for fetal development. Placental ischemia is thought to develop as a result of this abnormal cytotrophoblastic invasion; this has been proposed as leading to release of placental factors and imbalance of angiogenic factors, causing the widespread endothelial dysfunction which characterizes preeclampsia (Lee et al. 2007).

## Angiogenic factors

As research in the field of preeclampsia progresses, much of the attention in recent years has been focused on peptides related to angiogenesis. Angiogenesis, the development of new blood vessels from existing endothelium, is essential for normal placental development. Two of the angiogenic growth factors, vascular endothelial growth factor (VEGF) and placental growth factor (PlGF) are thought to contribute to normal trophoblastic proliferation and implantation (Thadhani et al. 2004), and it has been hypothesized that an imbalance in levels of these growth factors has a crucial role in preeclampsia. As normal pregnancy progresses, maternal VEGF expression is reduced (Ong et al. 2000a), but placental levels of mRNA-encoding VEGF have been shown to be much lower in women with preeclampsia compared to controls (Cooper et al. 1996). Similarly, maternal plasma levels of PlGF have been shown to be significantly reduced in the second trimester in women who went on to develop preeclampsia compared to controls (Kim et al. 2007). The use of anti-VEGF antibodies for systemic treatment in cancer has shown a dose dependant association with hypertension and proteinuria (Ostendorf et al. 2007), which may indicate that these factors have a role in the development of preeclampsia. Many recent studies have therefore concentrated on factors which antagonize VEGF and PlGF to assess their role in the development of preeclampsia. Two of the most extensively studied peptides, which are produced by the placenta, are soluble FMS-like tyrosine kinase (sFLT-1) and soluble endoglin.

## Soluble sFLT-1

Soluble FLT-1 (also known as soluble VEGF receptor 1 or sFLT-1) is a secreted splice variant of FLT-1. It binds to and neutralizes the angiogenic actions of VEGF and PlGF (Venkatesha et al. 2006) and is thought to be one of the key peptides involved in the development of preeclampsia. Maternal serum levels of sFLT-1 have been shown to be elevated in women with preeclampsia compared to controls (Koga et al. 2003, Salahuddin et al. 2007, Chaiworapongsa et al. 2004), to correlate with disease severity (Chaiworapongsa et al. 2004) and to decrease markedly following delivery (Koga et al. 2003). It has also been shown that that levels of sFLT-1 are increased in nulliparity (one of the main known risk factors for developing preeclampsia) when compared to multiparous women (Wolf et al. 2005). A series of studies by Maynard et al. (2003) revealed that mRNA of sFLT-1 is up-regulated in the placenta of women with preeclampsia, leading to increased systemic levels. Furthermore, these authors demonstrated that when a recombinant adenovirus encoding sFLT-1 was injected into pregnant rats, hypertension and proteinuria, as well as glomerular endotheliosis, one of the typical pathological lesions seen in preeclampsia, were observed. Further evidence for the placental origin of the elevated sFLT-1 was provided by Staff et al. (2005). Given that “delivery” includes both the fetus and the placenta, the group investigated whether fetal as well as maternal levels of sFLT-1 were elevated in preeclampsia. They found that although fetal levels of sFLT-1 (measured in cord blood) are elevated in preeclampsia, the maternal serum levels were 29-fold higher and concluded that there was no substantial fetal contribution to the elevated circulating maternal sFLT-1 levels seen in preeclampsia. Finally, serum sFLT-1 levels have been shown to be increased in women with preeclampsia superimposed upon systemic lupus erythematosis (Qazi et al. 2008) and glomerulonephritis (Masuyama et al. 2006).

## Soluble Endoglin

Another peptide that has been implicated in the pathogenesis of preeclampsia is soluble endoglin (sEng). Endoglin, a coreceptor for transforming growth factors *β*1 and *β*3 (Luft 2006), is highly expressed on endothelial cell membranes and syncytiotrophoblasts. Mutations in the gene encoding Eng are the underlying cause of hereditary haemorrhagic telangiectasia, a genetic condition characterized by atrioventricular malformations, epistaxis, and telangiectasiae (Venkatesha et al. 2006). In normal pregnancy, levels of sEng fall between the first and second trimesters, but in women who go on to develop preeclampsia, this reduction is blunted (Rana et al. 2007). Consistent with studies involving sFLT-1, it has also been demonstrated that levels of sEng are elevated in the sera of pregnant women with preeclampsia, correlate with disease severity, and fall after delivery (Venkatesha et al. 2006). A promising discovery in terms of predicting the condition was that levels of sEng are elevated several weeks before the development of clinical symptoms in women who developed preeclampsia; furthermore, in patients who developed preterm preeclampsia, the serum sEng levels are elevated (approximately twofold) as early as gestational weeks 17-20 (Levine et al. 2006). Rana et al. (2007) found that although levels of sEng and sFLT-1 were found to be elevated in the serum of preeclamptic women at 17-20 weeks gestation when compared to controls, the levels at 11-13 weeks were similar between cases and controls. Both sEng and sFLT-1 appear, therefore, to be important peptides in the pathogenesis of preeclampsia although, when used alone, do not appear to have a sufficiently high positive predictive value to be translated into routine clinical practice.

## Placental Protein 13

Placental protein 13 (PP-13) is a 32-kDa dimer protein, one of a group of proteins which are known to be highly expressed in the placenta. It has been prepared in recombinant form and is thought to be involved in placental implantation and maternal vascular remodeling (Nicolaides et al. 2006). During normal pregnancy, levels of PP-13 gradually increase, but abnormally low levels of PP-13 have been shown in weeks 11-13 gestation in women who went on to develop preeclampsia and FGR when compared with controls (Burger et al. 2004). A further study, analyzing maternal serum PP-13 levels at 9-12 weeks gestation, also found lower levels in women who went on to develop preeclampsia compared with controls (Chafetz et al. 2007). Combining maternal serum PP-13 to uterine artery Doppler studies early in pregnancy seems to improve ability to predict severe preeclampsia. Nicolaides et al. (2006) found that women who went on to develop preterm preeclampsia (requiring delivery before 34 weeks) had a higher median uterine artery pulsatility index, and a lower median serum PP-13 in the first trimester when compared to controls. Thus, they concluded that, for a 90% detection rate of the condition, using serum PP-13 for all women and Doppler studies in the 14% at highest risk, a false-positive rate of 6% could be achieved. PP-13, therefore, whether used alone or in combination with Doppler studies, appears to be a promising area for future research in this field.

## Pregnancy-Associated Plasma Protein A

Pregnancy-associated plasma protein A, (PAPP-A) is a large highly glycosylated protein complex produced by the developing trophoblast (Bersinger et al. 2003), which is used in many centers as a marker for Downs' syndrome. It has been shown to be responsible for the cleavage of insulin-like growth factor (IGF) binding proteins, which are inhibitors of IGF action, in several biological fluids (Laursen et al. 2001). PAPP-A was first shown to be elevated in the plasma of preeclamptic women nearly 30 years ago (Hughes et al. 1980). More recent studies have shown that although reduced first trimester serum levels of PAPP-A are associated with preeclampsia, levels are also low in other complications of pregnancy (Ong et al. 2000b, Yaron et al. 2002, Smith et al. 2002). It has been suggested that PAPP-A is more useful as a marker of FGR than of preeclampsia (Canini et al. 2008). In a recent paper, Spencer et al. (2008) described a small increase in likelihood ratio of developing preeclampsia with decreasing levels of PAPP-A. Although PAPP-A alone was not a good predictor for preeclampsia, they felt, similarly to PP-13, that sensitivity could be improved by combining with uterine artery Doppler studies.

## Insulin Resistance

Insulin resistance has long been implicated in the pathogenesis of preeclampsia. Carbohydrate metabolism is known to be altered in women with preeclampsia, whereas fasting insulin levels have also been shown to be elevated prior to the onset of disease (Spencer et al. 2005). Furthermore, types 1 and 2 diabetes, gestational diabetes, and polycystic ovarian syndrome are all well-established risk factors for the condition (Wolf et al. 2002).

Normal pregnancy is characterized by increased insulin secretion by the pancreatic *β* cells, and, following initially increased insulin sensitivity, there follows a progressive increase in insulin resistance throughout the second and third trimesters (Butte 2000). Sex-hormone-binding globulin (SHBG) is a glycoprotein produced by the liver which binds circulating estrogens and testosterone. Production of SHBG is inhibited by insulin; therefore, low levels of SHBG are associated with elevated insulin levels. As a result, studies have used low SHBG levels as a marker of insulin resistance in both cardiovascular disease (Sherif et al. 1998) and in preeclampsia (Carlsen et al. 2005, Spencer et al. 2005, Wolf et al. 2002). One study looking at first-trimester SHBG levels in 45 nulliparous women who developed preeclampsia found that levels were significantly reduced when compared with controls (Wolf et al. 2002). In contrast, however, a further study looking at SHBG levels at weeks 10-14 in 107 women with preeclampsia found no significant difference between women who went on to develop preeclampsia compared to controls (Spencer et al. 2005). This retrospective study included multiparous women, which may have confounded results. Another study found no difference at gestational weeks 17 or 33 between 29 cases of preeclampsia and controls (Carlsen et al. 2005), although the relatively small numbers of patients involved may be of relevance.

Adiponectin, an adipocyte-derived cytokine involved in carbohydrate and fat metabolism, is another protein whose levels are inversely correlated with insulin resistance. High concentrations of adiponectin have been shown to be protective against the development of type 2 diabetes (Lindsay et al. 2002), and serum levels of adiponectin have been shown to correlate with sEng levels in women with preeclampsia (Masuyama et al. 2007). It has been hypothesized that low serum adiponectin levels are associated with an increased risk of development of preeclampsia. D'Anna et al. (2006) examined serum adiponectin levels in the first trimester in women who subsequently developed preeclampsia. They found that levels were lower than those in controls, but the levels were significantly different between those who developed early-onset and late-onset symptoms, suggesting a different pathogenesis. Ramsay et al. (2003) found, in contrast to their initial hypothesis, that serum adiponectin levels in the third trimester are in fact higher in women with preeclampsia compared with controls, a finding which has been confirmed elsewhere (Fasshauer et al. 2008). It has been suggested that adiponectin forms part of the physiological response to preeclampsia, by improving insulin sensitivity (Fasshauer et al. 2008). It appears, therefore, that although insulin resistance has a prominent role in the pathogenesis of preeclampsia, its role in predicting the disease, and the best method for measuring it, are yet to be determined.

## Apolipoprotein E

One of the mechanisms by which preeclampsia has been postulated to develop is via abnormal lipid metabolism associated with oxidative stress. Women with preeclampsia have an abnormal lipid profile, with elevated concentrations of triglyceride-rich lipoproteins, which may contribute to endothelial dysfunction (Sattar et al. 1997). Apolipoprotein E (ApoE) is a major constituent of very low-density lipoproteins (VLDLs) whose role involves modifying inflammatory responses, and removal of excess cholesterol from the circulation via regulation of hepatic uptake (Belo et al 2004). The *APOE* gene on chromosome 19 has 3 common alleles, encoding 3 plasma ApoE isoforms, e2, e3, and e4. ApoE e4 is a known risk factor for familial Alzheimer's disease, whereas both e2 and e4 isoforms have been associated with abnormally high triglyceride and VLDL levels (Francoual et al. 2002). It has been postulated that ApoE levels and polymorphisms of its gene are associated with an increased risk of preeclampsia. Nagy et al. (1998), found a higher incidence of the ApoE e2 allele amongst women with preeclampsia compared to controls. Makkonen et al. (2001) studied 133 women with preeclampsia and, in contrast, found that none of the ApoE alleles were over represented when compared with controls, findings that have been confirmed elsewhere (Belo et al. 2004). The role of this avenue of investigation in the prediction of preeclampsia is therefore, at present, uncertain.

## Inhibin A and Activin A

Many studies have been reported using inhibin A and activin A as predictors of preeclampsia. Both are glycoproteins, are members of the transforming growth factor β family, and during pregnancy, are largely released by the fetoplacental unit. Inhibin A has an important endocrine role in the negative feedback of gonadotrophins, whereas activin A is thought to have activity in various biological tissues (Luisi et al., 2005; Muttukrishna et al. 2000). In normal pregnancy, concentrations of both hormones rise in the third trimester, and levels have been shown to be elevated approximately 10-fold in women with severe preeclampsia compared to controls (Muttukrishna et al. 1997). Second trimester levels of inhibin A have been shown to be elevated in both serum (Aquilina et al. 1999) and amniotic fluid (Kim et al. 2006) in women who went on to develop severe preeclampsia, and when measured at term, serum levels have been shown to correlate with preeclampsia severity (Zeeman et al. 2002). Interestingly, urinary activin A and inhibin A levels have also been found to be elevated in women with preeclampsia, as have uterine vein levels (Muttukrishna et al. 2006). The second trimester levels of both inhibin A and activin A have been reported to add significant prognostic information when measured in women with abnormal uterine artery Doppler studies (Florio et al. 2003). In contrast to the above, however, a study by Davidson et al. (2003) found that although second trimester levels of activin A were elevated in women who went on to develop preeclampsia, inhibin A levels were not elevated, findings confirmed by D'Anna et al. (2002). Studies using first trimester inhibin A (Roes et al. 2004) have also shown a low predictive value.

The cause of these rises in activin A and inhibin A is not yet fully understood; and whether these hormones have a role in the etiology of preeclampsia is not yet known. Further prospective studies, including measurement of urinary inhibin A, are clearly required.

## Genomics and Proteomics

A number of gene polymorphisms have been found to be associated with the risk of developing preeclampsia. A recent well-powered study into the genetics of preeclampsia, however, did not confirm significant associations between a single nucleotide polymorphisms in candidate genes and preeclampsia (GOPEC Consortium 2005). Recent advances in genotyping technology will facilitate genome-wide association studies in preeclampsia which will very likely result in novel candidate genes for the disorder. Results from studies into other polygenic disorders such as coronary artery disease and diabetes are promising (Wellcome Trust Case Control Consortium 2007), but investigators will have to follow strict rules to avoid false positive and underpowered negative results (NCI-NHGRI Working Group on Replication in Association Studies 2007).

**Figure 2:**
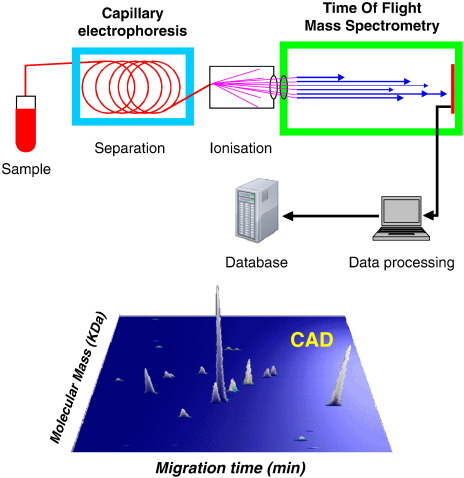
Capillary electrophoresis online coupled to mass spectrometry. Urine samples are prepared for analysis, polypeptides are separated by capillary electrophoresis and directly sprayed into electrospray ionization–time of flight mass spectrometry. Data are evaluated using specific software solutions. Each polypeptide is defined by its accurate mass and normalized CE migration time. Signal intensity serves as measure of the relative abundance. The data are stored as peak lists summarizing the information in a database. The process is demonstrated for use of CE–mass spectrometry to in the diagnosis of coronary artery disease. The lower panel shows a coronary artery disease-specific polypeptide pattern. The top panel is modified with permission from *Electrophoresis* 2007;28:1407-1417Sniehotta et al., 2007. Sniehotta et al: CE - a multifunctional application for clinical diagnosis. Electrophoresis. 2007. Volume 28. Pages 1407-1417. Copyright Wiley-VCH Verlag GmbH & Co. KGaA. Reproduced with permission.

Further advances are expected from proteomic research. Proteomics has been defined as “knowledge of the structure, function, and expression of all proteins in the biochemical or biological contexts of all organisms” (Kenyon et al. 2002). Comparing protein patterns between healthy patients and those with a disease has been increasingly used in recent years to discover markers of disease (biomarkers), which have a number of important roles in medical research. Proteomics can be used to improve early detection of disease, to develop new targets for therapeutic treatment, and to monitor response to treatment (Figure 1). To date, potential proteomic biomarkers have been reported for early diagnosis of cardiovascular disease (Zimmerli et al. 2008), renal transplant rejection (Wittke et al. 2005), urological cancers (Saito et al. 2005), and acute kidney injury (Zhou et al. 2006). Human urine is an ideal medium to study proteomics because of its ease of collection, and its relative stability. Normal human urine contains up to 150 mg of protein/24 hours and contains much useful information about the kidneys and urogenital tract; because urine is a filtrate of blood, pathological changes in the blood indicative of disease can be reflected in the urinary proteome (Gonzalez-Buitrago et al. 2007). Proteomics employs protein separation using two-dimensional gel electrophoresis, high-performance liquid chromatography, or capillary electrophoresis (CE), coupled online to a time-of-flight mass spectrometer (Figure 2). This allows the molecular weight of a single molecule to be measured and displayed according to its mass: charge ratio. Each peptide has a molecular “fingerprint,” and this technology allows simultaneous examination of several thousand peptides within minutes. Once a biomarker “signature profile” has been identified, it can be compared with healthy closely matched controls, allowing for a disease-specific biomarker to be identified.

It appears likely that the development of preeclampsia involves several different pathophysiological mechanisms, as evidenced by the diversity of the peptides that have been studied to date. Current research is driven by pathways that are known to be involved in the development of the condition, and one advantage of proteomics-based research is that many of these mechanisms can be integrated. In addition, since proteomics is a “hypothesis-free” research tool, it is likely that this line of investigation will open new avenues for potential biomarker discovery and for new diagnostic and preventative measures.

## Metabolomics

Metabolomics is a further area that has the potential to contribute significantly to future research in preeclampsia. In complement to studies examining the human genome and proteome, metabolomics can be defined as a “systematic study of the unique chemical fingerprints that specific cellular processes leave behind” (Daviss 2005). In common with proteomics, studies of the human metabolome can be carried out on routine samples of urine, plasma, or serum requiring minimal specialist preparation of samples. Metabolomics (also referred to as metanomics or metabonomics) has been used to characterize signature profiles for cardiovascular disease (Brindle et al. 2002), for Alzheimer's disease (Han et al. 2002), and for hypertension (Brindle et al. 2003). A study evaluating the ability of metabolomics in diagnosis of preeclampsia has been reported (Kenny et al. 2005). They discovered three metabolomic peaks which could be used with high sensitivity and specificity to differentiate women with preeclampsia from controls. In this study, the majority of samples were taken in late pregnancy, and so, the ability of these metabolomic peaks to predict preeclampsia before the onset of clinical disease remains uncertain. Although the group were, at that point, unable to identify the metabolites involved, it is likely that future research in this field will aid our understanding of this challenging condition.

## Conclusion

Although improvements in obstetric and neonatal care have led to a reduction in morbidity and mortality from preeclampsia, our ability to predict the condition has not improved significantly. We currently rely on “secondary prevention” of preeclampsia: women who have previously had the disease are closely monitored throughout pregnancy. The majority of women who develop preeclampsia, however, are only diagnosed once they have developed the full-blown manifestations of the condition, by which time treatment options are limited. Rather than being a separate condition, preeclampsia has been previously described as the extreme end of a maternal systemic response engendered by pregnancy itself (Redman and Sargent 2003). We have seen that many of the proposed biomarkers for preeclampsia are raised to a lesser extent in normal pregnancy, which will make discovery of accurate biomarkers for preeclampsia more difficult. Nevertheless, if signs of abnormal placental and endothelial dysfunction could be detected prior to the onset of clinical disease, they would represent an extremely attractive target for emerging therapeutic strategies. In addition, any such treatments would be most likely to be effective if they could be started in early gestation. In routine clinical practice, we currently screen for several conditions in early pregnancy, most of which have a far lower incidence than preeclampsia, and it is hoped that in time we will be able to do the same for preeclampsia.

An increased understanding of the molecular mechanisms underlying preeclampsia has led to several potential areas of investigation. In addition to studies mentioned above, novel biomarkers such as urine orosomucoid, an acute phase protein, show promise in early prediction of preeclampsia (Kronborg et al. 2007). To date, however, studies of potential biomarkers for predicting preeclampsia have involved relatively small numbers of patients, although many of the studies have ambiguous definitions of preeclampsia and unclear distinction of early and severe disease. It is unlikely that a single marker will prove to be an accurate predictive tool for preeclampsia; a combination of clinical information regarding history and risk factors along with urine or blood biomarkers in those at risk is more realistic. There is clearly a great need for development of predictive tools for preeclampsia; it is hoped that proteomics, metabolomics, and other techniques will allow us to develop biomarkers with high enough predictive and prognostic information to be translated into clinical practice. These developments could well be the key to improving care for women with this devastating condition.
